# Estimating need for alcohol treatment in Ireland using national treatment surveillance data

**DOI:** 10.1007/s11845-021-02788-9

**Published:** 2021-10-03

**Authors:** Anne Marie Carew, Derek O’Neill, Suzi Lyons, Bobby P. Smyth

**Affiliations:** 1 Health Research Board, Grattan House, 67-72 Lower Mount Street, Dublin, D02 H638 Ireland; 2 Department of Public Health & Primary Care, Trinity College Dublin, Dublin, Ireland

**Keywords:** Alcohol, Service planning, Treatment, Treatment need

## Abstract

**Background:**

International evidence indicates that about 10% of people with alcohol dependence will seek and commence treatment each year. Based upon Irish estimates of prevalence of dependence, a target of 690.0 treated cases per 100,000 population per annum is expected.

**Aims:**

This study analyses routine national surveillance data on alcohol treatment to measure how treatment need is being met.

**Methods:**

National treatment surveillance data on problem alcohol use collected by the National Drug Treatment Reporting System (NDTRS) were analysed. The study included cases resident in Ireland, aged 18–64 years entering treatment for alcohol use disorder (AUD) between 2015 and 2019 (*n* = 44,079). Treatment rates were calculated per 100,000 of the population. Descriptive and exploratory statistics were used to describe characteristics of cases treated.

**Results:**

National rate of treated AUD was 270 cases per 100,000 annually, with a rate of treated alcohol dependence of 165/100,000. There was a fivefold difference between the lowest and highest rates (119 cases per 100,000 in Meath versus 633 in Waterford). Drinking patterns indicate high levels of alcohol consumption and prolonged use prior to treatment. The use of other drugs alongside alcohol was common.

**Conclusions:**

Despite high rates of alcohol consumption and dependence, the rate of treatment entry nationally is sub-optimal, although there are wide geographic variations. There is a need to better understand the reasons for low treatment entry rates in Ireland for people with alcohol dependence. Monitoring and surveillance play a key role in measuring the successful efforts to reduce the harm of alcohol.

## Background

Alcohol use is the is the leading risk factor among those aged 15–49 years for both deaths and disability-adjusted life years globally [1]. Ireland has one of the highest per-capita alcohol consumption rates in the European Union, with levels of alcohol consumption and hazardous drinking patterns in Ireland projected to increase over the next decade [2]. The most recently available Irish figures estimate that among the general population, 6.9% are alcohol-dependent [3].

One complication when discussing treatment of alcohol problems relates to international differences in diagnostic categories. ICD 11 continues to distinguish two separate categories of problematic alcohol use, these being alcohol dependence and harmful alcohol use. DSM V has in recent years moved towards use of the single term of alcohol use disorder (AUD), this being rated on a spectrum from mild to severe [4].

Public health measures across the entire population are important in trying to reduce alcohol-related harm across Ireland [5], and high-quality accessible treatment for people with AUD is vital. Internationally, there is evidence that many people with an AUD fail to recognise their drinking as problematic [6–11]. This may be even more common in Ireland where just 16% of dependent drinkers in population samples recognise that they are drinking heavily [12]. Studies show that the annual rate of access to specialist treatment for AUD is about 10% [13]. With an estimated population prevalence of 6.9% alcohol-dependent, the target of treating 10% per annum suggests that there should be about 690 episodes of treated alcohol dependence per 100,000 adults each year in Ireland.

Although dependence and drinking frequency are strong predictors of treatment entry [14], the literature shows that there is a strong association between perceived need and use of services [15–21], with indications that 8.5–22.8% of people with substance use disorder perceiving a need for treatment [15, 21]. However, the proportion of people who both recognise that they need treatment and also take the step of seeking it out can be as low as 3% [22] to 6% [23].

In order to provide appropriate and adequate access to treatment for AUD in the population, it is imperative to understand the level of need and demand. Understanding the geographical distribution of treatment need is also crucial in establishing and maintaining adequate service levels. Some of the ways this can be achieved is through surveys looking at levels of alcohol consumption within the general population which can help predict levels of need [3, 12]. Treatment data can also be another useful source of information to assist in understanding the level of need. Not only is there a range of effective treatments available to address AUD, treatment is cost-effective [24, 25]. In Ireland, the range of treatment, rehabilitation, and recovery services are provided using a four-tier model of care [26] allowing the individual to access supports at the level of complexity that reflects their situation and needs [27], with more severe AUD directed to tier 3 (specialist outpatient addiction services) and tier 4 (residential) services. The majority of cases treated for AUD in Ireland attend outpatient settings [28].

The aim of this study was to determine the rate of treated AUD in the Irish population using routinely gathered health surveillance data to aid the planning and provision of adequate service provision.

## Methods

The study is a secondary analysis of routinely gathered health surveillance data from the Irish National Drug Treatment Reporting System (NDTRS). The NDTRS is a national register of addiction treatment in Ireland [29] and data is collected following the European TDI protocol, available at http://www.emcdda.europa.eu/publications/manuals/tdi-protocol-3.0_en. Treatment data are provided by statutory and non-statutory services, including outpatient services, residential centres, and prisons. Data on opioid substitution provided by general practitioners (GPs) is also included. The NDTRS is considered a comprehensive measure of treatment demand nationally and includes data from public, voluntary, and private treatment services in receipt of public funds [30]. NDTRS data coverage (numbers of admissions and treatment centres reporting data) is high [31], particularly in inpatient, outpatient, and low threshold settings [30]. Service providers collect data including client demographic and socioeconomic information, referral and assessment details, current problem drugs (up to four substances), treatment history, injecting risk behaviours, treatment interventions provided, and details of treatment outcome at the time of discharge or transfer to another service. The NDTRS records episodes, or cases of treatment as there is currently no national system-wide unique identifier in the Irish health system; in any given year, individuals may appear more than once if treated in different centres or if they return to treatment in the same centre. The study population included all cases resident in Ireland who entered treatment for problem use of alcohol in the period 2015–2019. Cases aged 18–64 years of age that reported alcohol as either their main or an additional problem drug were included (*n* = 44,079).

Rates of treatment entry were calculated per 100,000 of the population per annum based on census 2016 and use of published estimates for other years [32, 33]. A combination of descriptive, exploratory statistics was used to describe characteristics of cases treated for alcohol use. Appropriate measures were reported depending on data distributions. Data were managed and analysed in the Statistical Package for Social Sciences (SPSS), version 26.0 [34].

## Results

### Profile of cases entering treatment

A total of 44,079 cases entered treatment for problem alcohol use during the period 2015–2019. Of these, 34,836 (79.0%) cases had alcohol as a main problem, while 9243 (21.0%) cases had alcohol as an additional problem (Table 1). The majority of cases were male (67.4%). In cases where alcohol was the main problem drug, the age profile was older, with 1.3% aged less than 20 years compared to 8.0% of cases where alcohol was an additional problem drug. Almost one-in-four (24.4%) had ceased their education before their sixteenth birthday and unemployment was common. The most common source of referral was self-referral (40.5%), while 11.6% were referred to treatment by family or friends; 23.0% were health-related referrals (general practitioners/hospital or other medical settings, 10.7% were referred by social services and 2.9% were referred by the criminal justice system). The median age of first use of alcohol and other drugs was 15 years. A substantial proportion (41.8%) were new treatment cases and had never been treated for alcohol use previously. The majority (61.2%) were alcohol-dependent.

**Table 1:** Profile of cases seeking treatment for AUD

Variable	Alcohol as a main problem		Alcohol as an additional problem			Total	
	***N* = 34,836**	**%**	***N* = 9243**	**%**		***N* = 44,079**	**%**
**Sex**							
Male	22,711	65.2%	7001	75.7%		29,712	67.4%
Female	12,041	34.6%	2216	24.0%		14,257	32.3%
Unknown	84	0.2%	26	0.3%		110	0.2%
**Age group**							
15–19	93	1.3%	161	8.0%		254	2.8%
20–24	431	6.1%	407	20.3%		838	9.3%
25–29	702	10.0%	423	21.1%		1125	12.5%
30–34	911	13.0%	360	18.0%		1271	14.1%
35–39	1147	16.3%	305	15.2%		1452	16.1%
40–44	1079	15.4%	183	9.1%		1262	14.0%
45–49	949	13.5%	92	4.6%		1041	11.5%
50–64	1708	24.3%	70	3.5%		1778	19.7%
**Education**							
Primary level incomplete	520	1.5%	176	1.9%		696	1.6%
Primary level	4195	12.0%	1353	14.6%		5548	12.6%
Junior cert	10,114	29.0%	3403	36.8%		13,517	30.7%
Leaving cert	11,644	33.4%	2771	30.0%		14,415	32.7%
Third level	5034	14.5%	631	6.8%		5665	12.9%
Never went to school	64	0.2%	9	0.1%		73	0.2%
Not known	3265	9.4%	900	9.7%		4165	9.4%
**Age ceased education**							
Before 16 years	8068	23.2%	2695	29.2%		10,763	24.4%
**Employment**							
Employed	9620	27.6%	1866	20.2%		11,486	26.1%
Unemployed	19,355	55.6%	5450	59.0%		24,805	56.3%
Student	565	1.6%	265	2.9%		830	1.9%
Other	4730	13.6%	1426	15.4%		6156	14.0%
Not known	566	1.6%	236	2.6%		802	1.8%
**Living where**							
Stable accommodation	29,017	83.3%	7013	75.9%		36,030	81.7%
Homeless	3126	9.0%	808	8.7%		3934	8.9%
Other unstable accommodation	937	2.7%	259	2.8%		1196	2.7%
Prison	711	2.0%	707	7.6%		1418	3.2%
Residential care/halfway house	648	1.9%	320	3.5%		968	2.2%
Not known	397	1.1%	136	1.5%		533	1.2%
**Living arrangements**							
Alone	8170	23.5%	1113	12.0%		9283	21.1%
Parents and family	9321	26.8%	3987	43.1%		13,308	30.2%
Friends	838	2.4%	261	2.8%		1099	2.5%
With partner	3462	9.9%	490	5.3%		3952	9.0%
Partner and child	5863	16.8%	964	10.4%		6827	15.5%
Alone with child	2252	6.5%	358	3.9%		2610	5.9%
Other	4433	12.7%	1876	20.3%		6309	14.3%
Not known	497	1.4%	194	2.1%		691	1.6%
**Dependent children**							
Living with dependent children	8115	23.3%	1322	14.3%		9437	21.4%
**Region**							
Dublin	8745	25.1%	2863	31.0%		11,608	26.3%
Rest of Ireland	25,894	74.3%	6328	68.5%		32,222	73.1%
Ireland unknown	197	0.6%	52	0.6%		249	0.6%
**Source of referral**							
Self	14,214	40.8%	3654	39.5%		17,868	40.5%
Family and friends	3992	11.5%	1127	12.2%		5119	11.6%
Other drug treatment centre	2185	6.3%	1180	12.8%		3365	7.6%
General practitioner	4399	12.6%	519	5.6%		4918	11.2%
Hospital or other medical source	4591	13.2%	604	6.5%		5195	11.8%
Social services	3691	10.6%	1017	11.0%		4708	10.7%
Court/probation/police	873	2.5%	422	4.6%		1295	2.9%
Other	668	1.9%	650	7.0%		1318	3.0%
Not known	223	0.6%	70	0.8%		293	0.7%
**Extent of drinking problem**							
Hazardous	3307	9.5%	2347	25.4%		5654	12.8%
Harmful	5894	16.9%	2663	28.8%		8557	19.4%
Dependent	23,808	68.3%	3423	37.0%		27,231	61.8%
Not known	1827	5.2%	810	8.7%		2637	6.0%
**Consumption on a typical drinking day**	Median (25th,75th percentile)						
Number of standard drinks (among those consuming alcohol in the month prior to treatment)	16 (6, 36)		15 (5, 32)			16 (6, 35)	
**Duration of use**	Median (25th,75th percentile)						
Years prior to first-treatment	23 (6, 42)		13 (4, 30)			21 (5, 42)	
**Treatment status**							
New alcohol treatment cases	15,828	45.4%	2612		28.3%	18,440	41.8%
**Polydrug use**							
More than one problem drug	7056	20.3%	9243		100.0%	16,299	37.0%
**Problem drug use**	*Additional problem drugs^ (n* = *7056)*		*Main problem drug where alcohol was an additional problem*				
Cannabis	4083	57.9%	2276		24.6%		
Cocaine	3082	43.7%	2825		30.6%		
Benzodiazepines	1728	24.5%	1183		12.8%		
Opioids	1005	14.2%	2409		26.1%		
Ecstasy	659	9.3%	69		0.7%		
Amphetamines	258	3.7%	68		0.7%		
NPS	104	1.5%	67		0.7%		
Z drugs	132	1.9%	63		0.7%		
Other drugs	271	3.8	283		3.1		
**Waiting times for treatment**	Median (25th,75th percentile)						
Referral to treatment start (days)	4 (0, 54)		4 (0, 58)			4 (0, 55)	
	Median (25th,75th percentile)						
**Age first alcohol use**	15 (12, 21)		14 (11, 18)			15 (12, 21)	

^Cases where alcohol was the main problem drug. Up to four additional problem drugs may be reported

Consumption levels were high with half of episodes consuming 12 standard drinks on a typical drinking day. Drinking patterns indicate prolonged alcohol use prior to seeking treatment (median duration of use prior to first treatment = 21 years). Overall, 37.0% reported problem use of at least one other drug. Among those with alcohol as a main problem, 20.3% reported problem use of other drugs. The most common additional problem drugs were cannabis, followed by cocaine and benzodiazepines. For those cases for whom alcohol was an additional problem, cocaine, opioids, and cannabis were the most common main problem drugs. The median time between referral and treatment start was 4 days.

### Rates of treatment

**Fig. 1:**
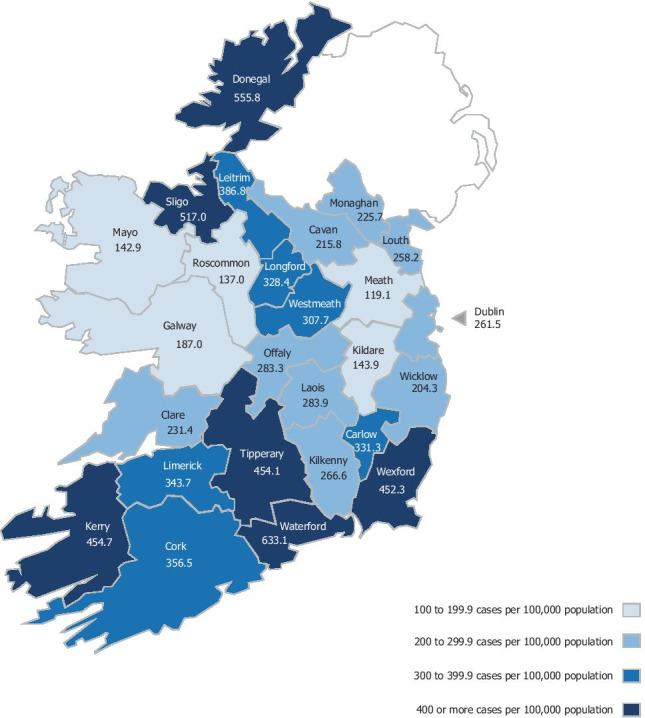
Average annual prevalence of treated AUD per 100,000 population, by county of residence (NDTRS 2015–2019; CSO 2016)

In the period 2015–2009, national annual rate of treated AUD (all cases) was 270 cases per 100,000 of the 18–64-year-old population. When looking specifically at those who were identified as alcohol-dependent when treated, the rate drops to 165/100,000 per annum. The analysis by county of residence (Fig. 1) shows that in the period 2015–2019 the treatment rates were highest in Waterford, Donegal, and Sligo (with more than 500 cases per 100,000 per annum), and lowest in Meath, Roscommon, Mayo, and Kildare (with less than 150 cases per 100,000 per annum). There was a fivefold difference between the lowest and highest treatment rates (119 per 100,000/annum in Meath versus 633 in Waterford). A total of twelve counties had treatment rates which were below the national rate, while in counties Meath and Roscommon, the rate was less than half that of the national rate.

## Discussion

This study indicates a national rate for treated AUD is 270 cases per 100,000 population per annum. If data is confined to treated cases with alcohol dependence, the rate of treatment entry is just 165/100,000 per annum. These figures fall well short of the identified target of 690 treatment episodes for dependent drinkers per 100,000 adults per annum. If the population prevalence of alcohol dependence in adults is correct, these findings suggest that only 2–3% of those with dependence are entering treatment each year. The literature recognises that there are ‘hidden’ populations, along with many reasons for seeking treatment [14] or not seeking treatment [14, 35–37]. Nonetheless, studies show that there is a strong association between perceived need and use of services [15–21], with indications that 8.5–22.8% of people with substance use disorder perceiving a need for treatment [15, 21].

Our Irish treatment target (690/100,000 population) is based on the best estimate of the population prevalence and on rates of treatment engagement in the international literature. The target is lower in comparison to similar rates reported in the literature [38, 39]. However, a recent study from the USA found that AUD was the most common substance use disorder and among those with AUD past-year treatment use was 6.3% (CI: 5.49–7.18) [23], while earlier studies from other countries report rates of below or near 10% [40–43]. We opted to set the target for Ireland at 10% in this study as there are differences in study methodology or inclusion criteria. For example, some treatment entry figures in international studies include attendance at services such as alcoholics anonymous and primary care GPs which are not reported in NDTRS alcohol treatment figures. Nonetheless, our target figure for treated alcohol dependence for Ireland is not unrealistic given that it is below that reported for other countries.

Although the national rate of treatment entry was lower than the target rate, there is substantial geographical variation. There was a fivefold difference in rates contrasting the counties with the highest rates of treatment entry with the lowest rates. According to the latest available survey of the population, the prevalence of past-year alcohol use by people aged over 15 years ranged between 67 and 83% across Ireland [44]. Therefore, some regional variation in levels of alcohol consumption in this study is expected but it is unlikely that this variation is the sole cause of such large differences in treatment entry. In fact, the area with the lowest last year drinking prevalence of 67% has the some of the highest rates of treatment entry (Donegal, Sligo, Leitrim) [44].

It is possible that the variance in treatment availability by county may be influenced by local service provider participation in the NDTRS. There are high levels of service provider coverage in the NDTRS [31]; nonetheless, not all eligible treatment services participate. Over the last 6 years, the number of services participating in the NDTRS has remained steady at approximately 600 services [30]. However, NDTRS coverage varies by HSE CHO area and this may explain some of the observed variation in rates. For example, coverage of services in CHO 3 (Clare, Limerick, and North Tipperary/East Limerick areas) is below the national average with just 65% of treatment services located in this region actively participating in the NDTRS, while just 59% of services located in CHO 6 (Wicklow, Dun Laoghaire, and Dublin South East areas) actively participate in the NDTRS [30]. It is encouraging that treatment rates in some counties were approaching the target rate and indicates that it is possible to attain acceptable rates of treatment entry in an Irish context. This study cannot shed light on why those counties achieved such success while others appear to be failing to meet to the needs of adults with alcohol dependence. There are substantial benefits to routine alcohol screening, early intervention, and referral to treatment [45]. Alcohol treatment is effective in reducing harms and it is cost-effective [24, 25]. In order to reduce alcohol-related harms and support recovery, the obstacles to treatment entry should be better understood and treatment capacity increased where it is a limiting factor in treatment entry. Planning projections for clinical services involved in the treatment of alcohol use disorders should be informed by population need rather than historic levels of provision [38].

Past evidence indicates that most dependent drinkers in Ireland fail to describe their own alcohol use as heavy drinking [12], and many Irish drinkers have a poor understanding of low-risk drinking patterns. Consequently, it seems highly likely that many with alcohol dependence have not even contemplated a need for treatment [9]. Efforts to improve understanding of harmful patterns of drinking across the population may have the double benefit of reducing use and simultaneously increasing treatment seeking among the subset of drinkers who are dependent.

A person’s pattern of drinking is an important determinant of alcohol-related harm [12]. The NDTRS data shows that alcohol consumption levels were high, showing frequent risky single occasion drinking (RSOD), or binge drinking. Given that two-in-three cases were already alcohol-dependent before seeking treatment, more needs to be done to understand and address these delays. This drinking pattern is associated with a number of negative health, social, and economic consequences including liver cirrhosis, various types of cancer, increased likelihood of driving under the influence of alcohol, intentional self-harm, injury, and risky sexual behaviours [46–49]. These results also suggest that further work is required to educate the Irish public on low-risk drinking limits and it may be useful to introduce low-risk daily limits.

The median age of first use of alcohol and other drugs was 15 years which is not surprising given the decline in age of first drinking over past 40 years in Ireland [50]. This supports the need to delay the initiation to drinking among young people to reduce alcohol-related harm particularly as it is a known risk factor for later alcohol dependence [51]. NDTRS data will enable policymakers to measure the impact of any policy changes and may allow health service planners to allocate appropriate resources to the treatment of problem alcohol use.

The association between alcohol and additional problem substances was examined as information about the combinations of substances used is important in terms of individual clients’ care plans. Over a third of all cases reported problems with other drugs. Cannabis was the most commonly reported substance used alongside alcohol while cocaine was the second most commonly reported substance used alongside alcohol. This is not unexpected as cannabis is the most common drug used in the population as per the general population survey, and the general population study also showed the relationship between cannabis and alcohol use [52]. A recent study of substance use disorder comorbidity combinations shows that not only does alcohol feature in the four most common comorbidity combinations, alcohol and cannabis use disorder was also the most common comorbidity combination and this combination had the lowest receipt of treatment [23]. These data highlight the association between alcohol and other substances and the need for an integrated approach to the management of the full range of substance use disorders in Ireland.

### Strengths and limitations

Data on treated alcohol cases from the NDTRS continue to provide valuable information which allows alcohol and drug services to understand the extent of the problem, the personal and substance-using characteristics of those seeking treatment, and trends in treatment seeking over time. These data enable health service planners to allocate appropriate resources to the treatment of problem alcohol use. Population characteristics may not generalise beyond the treatment seeking population. Within the NDTRS, it is possible that individuals may appear more than once if they have more than one treatment within a single calendar year. In the absence of a national system-wide unique identifier in the Irish health system, it is not possible to accurately distinguish between cases and individuals. It also does not include those who were treated via entry into mutual self-help programmes such as AA or treated by their GPs only.

Despite these high levels of service provider coverage by NDTRS [31], not all treatment services participate in the system. Therefore, it is possible that the variance in treatment availability by county may be influenced by local service provider participation in the NDTRS. Notwithstanding the Health Research Board’s best efforts and also the requirement for all publicly funded services to make NDTRS returns [53], some alcohol treatment services managed by the addiction and mental health services do not participate. For this reason, coverage of cases remains incomplete in counties Dublin, Galway, Mayo, the Midlands (Laois, Offaly, Longford, Westmeath), and North East (Cavan, Monaghan, Louth, and Meath). Rates may also be affected by the availability of alcohol-specific services. It may therefore be assumed that the data presented in this paper underestimate the true extent of treated AUD in Ireland. In Ireland, those with substance use disorder may access inpatient treatment in psychiatric hospitals and these cases are not recorded by the NDTRS. In 2019, 1090 cases admitted to psychiatric inpatient facilities with an alcohol disorder, with trends over time indicating an overall decline in such admissions, which only slightly alters the proportion of those with AUD receiving treatment [54]. Nonetheless, data regarding outpatient treatments from such psychiatric hospitals is not returned to the NDTRS, despite best efforts of the Health Research Board to secure such data.

### Implications

There is a need to urgently address the relatively low rate of entry into treatment by people with alcohol dependence in Ireland. Efforts must be undertaken to better understand this service failure. There seem to be issues with problem recognition among affected individuals and there may also be unknown barriers and obstacles to treatment seeking among those who perceive a need for treatment. The general population survey of alcohol and drug use may offer an opportunity to explore these issues in the future.

As stated in Ireland’s current National Drugs Strategy, there is a need for all treatment providers to fully participate in the NDTRS system, so that the real extent of under-treatment can be comprehensively examined. There is substantial geographical variability in the recorded rate of treatment entry. There is a need for services in counties with low rates of treatment entry to learn from the successes of those in locations with acceptable levels of treatment entry.

## Conclusions

The proportion of people with alcohol dependence who enter treatment each year is low by international standards, and there is wide and unexplained geographical variation in rates of treatment attendance. There appears to be a need to better plan the provision of treatment services, so treatment is available where and when people seek it, with opportunities to learn from locations with higher rates of treatment entry. Strategies for early identification of AUD, such as the Making Every Contact Count initiative across all healthcare settings, may result in greater treatment seeking in the near future.

## Data Availability

The data used is available on request from the Health Research Board.

## Code availability

Data were managed and analysed in the Statistical Package for Social Sciences (SPSS), version 26.0. Code is available on request.
